# Impaired degradation followed by enhanced recycling of epidermal growth factor receptor caused by hypo-phosphorylation of tyrosine 1045 in RBE cells

**DOI:** 10.1186/1471-2407-12-179

**Published:** 2012-05-16

**Authors:** Anping Gui, Akira Kobayashi, Hiroaki Motoyama, Masato Kitazawa, Michiko Takeoka, Shinichi Miyagawa

**Affiliations:** 1 First Department of Surgery, Shinshu University School of Medicine, Asahi 3-1-1, Matsumoto, Nagano, 390-8621, Japan

**Keywords:** Cholangiocarcinoma, RBE cells, EGFR, Tyrosine 1045, Down-regulation, Recycling, Target

## Abstract

**Background:**

Since cholangiocarcinoma has a poor prognosis, several epidermal growth factor receptor (EGFR)-targeted therapies with antibody or small molecule inhibitor treatment have been proposed. However, their effect remains limited. The present study sought to understand the molecular genetic characteristics of cholangiocarcinoma related to EGFR, with emphasis on its degradation and recycling.

**Methods:**

We evaluated EGFR expression and colocalization by immunoblotting and immunofluorescence, cell surface EGFR expression by fluorescence-activated cell sorting (FACS), and EGFR ubiquitination and protein binding by immunoprecipitation in the human cholangiocarcinoma RBE and immortalized cholangiocyte MMNK-1 cell lines. Monensin treatment and Rab11a depletion by siRNA were adopted for inhibition of EGFR recycling.

**Results:**

Upon stimulation with EGF, ligand-induced EGFR degradation was impaired and the expression of phospho-tyrosine 1068 and phospho-p44/42 MAPK was sustained in RBE cells as compared with MMNK-1 cells. In RBE cells, the process of EGFR sorting for lysosomal degradation was blocked at the early endosome stage, and non-degradated EGFR was recycled to the cell surface. A disrupted association between EGFR and the E3 ubiquitin ligase c-Cbl, as well as hypo-phosphorylation of EGFR at tyrosine 1045 (Tyr1045), were also observed in RBE cells.

**Conclusion:**

In RBE cells, up-regulation of EGFR Tyr1045 phosphorylation is a potentially useful molecular alteration in EGFR-targeted therapy. The combination of molecular-targeted therapy determined by the characteristics of individual EGFR phosphorylation events and EGFR recycling inhibition show promise in future treatments of cholangiocarcinoma.

## Background

Cholangiocarcinoma is the second most common primary malignancy of the liver whose only therapeutic option is radical surgical resection or hepatectomy [1]. As the 5-year overall survival rate with curative resection (R0 resection) is reported to be 30.4% for intrahepatic cholangiocarcinoma [2], effective adjuvant therapy is needed to improve disease prognosis. Molecular-targeted treatment is superior to traditional chemotherapy through its ability to selectively suppress cancer cells. Overexpression [3], gene amplification [4], and mutation [5] of epidermal growth factor receptor (EGFR) have all been associated with the tumorigenesis and progression of cholangiocarcinoma, and thus EGFR and its molecular transducers are thought to be ideal therapeutic targets for cholangiocarcinoma treatment [6,7]. However, further understanding of the mechanism of aberrant EGFR signaling is needed to refine molecular targeted therapy due to the limited response rate of cholangiocarcinoma to EGFR-targeted therapy in clinical trial [8].

EGFR has important functions in cell proliferation, survival, migration, and differentiation via the activation by several distinct ligands, which include EGF, transforming growth factor-alpha and heparin-binding EGF [9]. The receptor stimulates numerous signal transduction cascades, such as those for mitogen-activated protein kinase (MAPK), phosphoinositol kinase, the anti-apoptotic kinase Akt, and several transcriptional regulators [10]. Aberrant EGFR activity has been shown to play a key role in the development and growth of various types of cancer cells [11,12]. To date, several mechanisms involving abnormal activation of EGFR have been reported, including increased production of ligands, increased levels of EGFR protein, EGFR mutations giving rise to constitutively active variants, defective down-regulation of EGFR, and cross-talk with heterologous receptor systems [13].

Down-regulation of EGFR by endocytosis and degradation is the major negative regulatory mechanism of attenuating EGFR signaling activation [14]. Upon ligand binding, cell surface EGFR is endocytosed into the cytosol and sequestered in a sorting/early endosome for either recycling or degradation [15]. Ubiquitination mediated by the E3 ubiquitin ligase Cbl is a key post-translational protein modification in EGFR endocytosis in porcine aortic endothelial (PAE), Hela, and laryngeal carcinoma cell line Hep2 cells [16,17], as well as in EGFR degradation in Chinese hamster ovary (CHO) and human embryonic kidney (HEK) cell line 293 T cells [18,19]. In a related report, Cbl mutation and reduced EGFR ubiquitination inhibited early endosome fusion and EGFR degradation in HEK 293 cells [20].

The present study demonstrated impaired degradation and enhanced recycling to the plasma membrane of EGFR under EGF stimulation in RBE cells. Since hypo-phosphorylation of Tyr1045 and a diminished association of c-Cbl and EGFR were considered as the responsible mechanisms, upregulation of Tyr1045 phosphorylation might be a useful alteration in EGFR-targeted therapy.

## Methods

### Materials and antibodies

Human epidermal growth factor (hEGF) was purchased from Roche Diagnostics GmbH (Mannheim, Germany). Goat anti-EGFR polyclonal IgG (1005 G), mouse anti-EGFR monoclonal IgG2 (528), mouse anti-Ub monoclonal IgG1 (6 C1), rabbit anti-Cbl polyclonal IgG (C-15), and mouse anti-LAMP-1 monoclonal IgG1 (H4A3) were purchased from Santa Cruz Biotechnology (Santa Cruz, CA). Mouse anti-β-actin monoclonal antibody (AC-15) was purchased from Sigma Aldrich (St. Louis, MO). Rabbit anti-phospho-EGF receptor (Tyr1068) IgG (D7A5), rabbit anti-phospho-EGF receptor (Tyr1045) antibody, rabbit anti-p44/42 MAPK IgG (137 F5), rabbit anti-phospho-p44/42 MAPK IgG (20 G11), and rabbit anti-EEA-1 IgG (C45B10) were purchased from Cell Signaling Technology (Danvers, MA). All oligonucleotides used in this study were synthesized by Sigma Aldrich.

### Cell culture and treatment

The immortalized human cholangiocyte MMNK-1 cell line [21,22] was provided by the Department of Surgery of Okayama University School of Medicine. The human intrahepatic cholangiocarcinoma RBE cell line was provided by the Cell Resource Center of Tohoku University (Sendai, Japan). Both cell lines were maintained in RPMI 1640 medium (Invitrogen, Tokyo, Japan) containing 10% FBS supplemented with penicillin and streptomycin at 37°C in 5% CO_2_. For cell starving assays, 60–80% confluent cell cultures were starved in RPMI 1640 medium containing 0.1% FBS for 12–16 hr before experiments. To induce EGFR activation, cells were incubated with EGF (100 ng/ml) in starving medium for the indicated time intervals. For long term incubation, EGF was renewed every 15 min [23]. For recycling inhibition, monensin (Sigma Aldrich) treatment was performed as described previously [24].

### Immunoblotting

Cells were lysed with 1 ml of ice-cold lysis buffer [0.5% Triton X-100, 50 mM Tris, pH7.5, 150 mM sodium chloride, 1 mM EDTA, 1 tablet/10 ml of Complete Mini, EDTA-free proteinase inhibitor cocktail, and 1 tablet/10 ml of PhosStop phosphatase inhibitor cocktail (Roche Diagnosis GmbH)]. Cell lysates were subjected to SDS-PAGE and transferred to PVDF membranes. Bands were scanned with a Canoscan 8400 F (Canon, Tokyo, Japan) and the optical intensity was quantified by ImageJ software (National Institutes of Health, Bethesda, MD).

### Immunoprecipitation

Total cell lysates were incubated with anti-EGFR antibody (528) for 4 hr before further incubation with protein-G sepharose beads (Amersham Biosciences, NA, UK) for 2 hr at 4°C. The immunoprecipitate was eluted and subjected to SDS-PAGE and immunoblotting. Anti-ubiquitin blotting was done as described previously [25]. Membranes were submerged in distilled water and autoclaved for 30 min at 121°C before immunoblotting.

### Immunofluorescence and confocal fluorescence microscopy

Cells were seeded onto sterile glass coverslips and fixed with 4% paraformaldehyde for 15 min before being blocked and permeabilized in blocking solution (10% normal goat serum and 0.3% Triton-X 100 in PBS). The coverslips were then incubated with appropriate primary and secondary antibodies for 1 hr at room temperature, respectively. Images were taken by confocal laser-scanning microscopy with a Leica TCS SP2 (Leica Microsystems, Heidelberg, Germany). Colocalization of EGFR with EEA-1 or LAMP-1 was quantified using ImageJ software and the Colocalization Highlighter plugin (P. Bourdoncle, Institute Jacques Monod, Service Imagerie, Paris, France) as described previously [26].

### Flow cytometry

Live cell immunostaining and flow cytometry were performed as described elsewhere [25]. All samples were examined by immunostaining in duplicate using anti-EGFR antibody (528) and Mouse IgG2 negative control antibody (Dako, Carpinteria, CA). Cells were then incubated with Alexa Fluor 488 goat anti mouse IgG secondary antibody for 30 min. Flow cytometry was performed and analyzed with a FACS Calibur machine (Becton Dickinson, Franklin Lakes, NJ) using CellQuest software (Becton Dickinson). For each sample, the mean fluorescence intensity (MFI) value of the negative control was subtracted from the MFI value of the anti-EGFR antibody to calculate the specific EGFR MFI staining.

### Real-time PCR

Total cell RNA was extracted with an RNeasy Mini kit (Qiagen, Hilden, Germany) and reverse transcribed to cDNA with a Takara PrimeScript RT reagent kit (Takara Bio, Shiga, Japan). Real-time PCR was performed with Takara SYBR Premix Ex Taq (Takara Bio) using an Eppendorf Mastercycler ep realplex (Eppendorf, Tokyo, Japan). Expression of β-actin in each sample was quantified for standardization of RNA amount. Primers used for human EGFR and β-actin are described elsewhere [27] and are listed in Table 1.

**Table 1:** Primers for PCR, real-time PCR and Oligonucleotides for Rab11a gene silence

EGFR exon 18 ~ 21	Sense	cctaagatcccgtccatcgcc
	Antisense	cactttgcctccttctgcatggta
EGFR exon 22 ~ 27	Sense	ttgggctggccaaactgctgg
	Antisense	caggcactgggaggaaggtgt
EGFR exon 28	Sense	ccacaggcgccttgactgagga
	Antisense	gcaacttcccaaaatgtgcccg
	Sense No.1	agtcgggctctggaggaa
	Antisense No.1	ggcagttctcctctccg
	Sense No.2	ctgtgcaacgtggagagc
EGFR	Antisense No.2	ccatctcatagctgtcgg
exon 2 ~ 16 (5 pairs)	Sense No.3	cccaccacgtaccagatg
	Antisense No.3	ccatgttgcttggtcctgcc
	Sense No.4	gctgattcaggcttggcc
	Antisense No.4	ctcaccctccagaagcttgc
	Sense No.5	ggtctgccatgccttgtg
	Antisense No.5	ggcccattcgttggacag
EGFR (real-time PCR)	Sense	gcacctacggatgcactgg
	Antisense	ggcgatggacgggatctta
β-actin (real-time PCR)	Sense	acgtggacatccgcaaagac
	Antisense	caagaaagggtgtaacgcaacta
Rab11a (real-time PCR)	Sense	ggcacagatatgggacacagc
	Antisense	aaggcacctacagctccacg
Rab11a siRNA	Sense	aaugucagacagacgcgaaaatt
	Antisense	uuuucgcgucugucugacauutt

### RNA interference analysis

Rab11a was depleted with siRNA oligonucleotides as described previously [28]. siRNA was transfected into RBE cells using Lipofectamine 2000 (Life technologies, Gaithersburg, MD). Transfected cells were incubated for another 48 hr before immunofluorescence analysis. Rab11a expression in mock- and siRNA-transfected RBE cells was quantified by real-time PCR.

### Mutation analysis of the EGFR gene

For mutation analysis of the EGFR gene, direct cDNA sequencing following PCR amplification was conducted using a BigDye Terminator v3.1 Cycle sequencing kit (Applied Biosystems, Foster City, CA) and was analyzed with an ABI Prism 3100 Sequencer (Applied Biosystems). The primers for PCR amplification are listed in Table 1.

### Statistical analysis

Results were expressed as mean ± standard error of the mean (mean ± SE). Differences in results were tested with a two-tailed Mann–Whitney *U* test (StatView, Cary, NC). A p < 0.05 was considered to be statistically significant.

## Results

### EGFR degradation was impaired upon EGF stimulation in RBE cells

**Figure 1:**
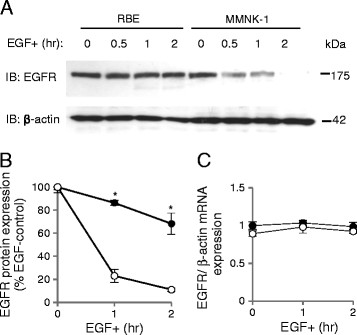
**Epidermal growth factor receptor (EGFR) degradation upon EGF stimulation in RBE and MMNK-1 cells.** (**A**) EGFR expression before and after 0.5, 1, and 2 hr of EGF treatment as detected by Western blotting. (**B**) Quantification of EGFR expression after 1 and 2 hr of EGF stimulation in RBE cells (closed circles) and MMNK-1 cells (open circles) from Western blotting. Values are standardized to the optical intensity of β-actin and presented as the percentage value of the EGFR expression value of RBE or MMNK-1 cells without EGF stimulation. *p < 0.05. (**C**) EGFR mRNA expression in RBE cells (closed circles) and MMNK-1 cells (open circles) before and after 1 and 2 hr of EGF stimulation as analyzed by RT-PCR. Values are standardized to β-actin mRNA expression and presented as the relative value to EGFR mRNA expression of RBE cells without EGF stimulation. All results shown are representative of four independent experiments.

We first assessed EGF-induced degradation of EGFR in RBE and MMNK-1 cells by Western blotting (Figure 1A). EGFR degradation was impaired in RBE cells compared with MMNK-1 cells (Figure 1A, B). After 1 hr of EGF stimulation, the expression of EGFR was 86.3 ± 2.2% of that of baseline in RBE cells, as compared with 23.1 ± 5.6% in MMNK-1 cells (p < 0.05, n = 4, Figure 1B). After 2 hr of EGF stimulation, expression of EGFR was 68.2 ± 9.2% in RBE cells versus only 11.1 ± 1.4% in MMNK-1 cells (p < 0.05, n = 4, Figure 1B). We also evaluated EGFR gene expression in RBE and MMNK-1 cells before and after EGF stimulation, which revealed no significant differences between these two cell lines before or after 1 or 2 hr of EGF stimulation (Figure 1C).

### EGFR downstream signaling was sustained upon EGF stimulation in RBE cells

**Figure 2:**
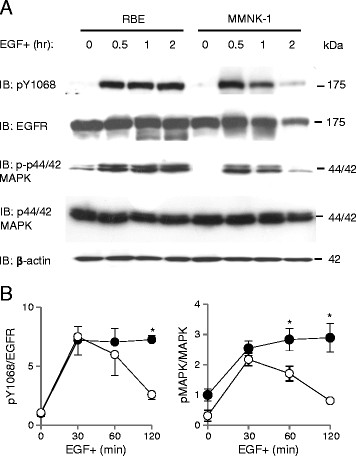
**Activation of EGFR signaling pathways upon EGF stimulation in RBE and MMNK-1 cells.** (**A**) Expression of pY1068, total EGFR, phospho-p44/42 MAPK (p-p44/42 MAPK), and total p44/42 MAPK before and after 0.5, 1 and 2 hr of EGF stimulation in RBE cells (closed circles) and MMNK-1 cells (open circles) as detected by Western blotting. (**B**) Left: quantification of pY1068 expression normalized to total EGFR. All values are presented as the relative value to the value of pY1068/total EGFR in RBE cells without EGF stimulation. Right: quantification of p-p44/42 MAPK expression normalized to total p44/42 MAPK. All values are presented as the relative value to the value of p-p44/42 MAPK/total p44/42 MAPK in RBE cells without EGF stimulation. *p < 0.05. All results shown are representative of three independent experiments.

To investigate the impact of impaired degradation of EGFR on EGFR-signaled pathways, we studied the expression of phosphorylated EGFR (pY1068) and downstream phosphorylated p44/42 MAPK (p-p44/42 MAPK) (Figure 2A). The expression of pY1068 persisted in RBE cells while a marked decrease of pEGFR was witnessed in MMNK-1 cells following 2 hr of EGF stimulation (7.2 ± 0.3 vs. 2.6 ± 0.4 folds of pY1068/total EGFR of RBE cells before EGF stimulation)(p < 0.05, n = 3, Figure 2B). Likewise, p-p44/42 MAPK persisted in RBE cells, but decreased significantly in MMNK-1 cells after 1 (2.8 ± 0.4 vs. 1.7 ± 0.2 folds of p-p44/42 MAPK/total p44/42MAPK of RBE cells before EGF stimulation) and 2 hr (2.9 ± 0.5 vs. 0.8 ± 0.0 folds of p-p44/42 MAPK/total p44/42MAPK of RBE cells before EGF stimulation) (p < 0.05, n = 3, Figure 2B) of EGF stimulation.

### Post-endocytic trafficking of EGFR was blocked at the early endosome stage in RBE cells

**Figure 3:**
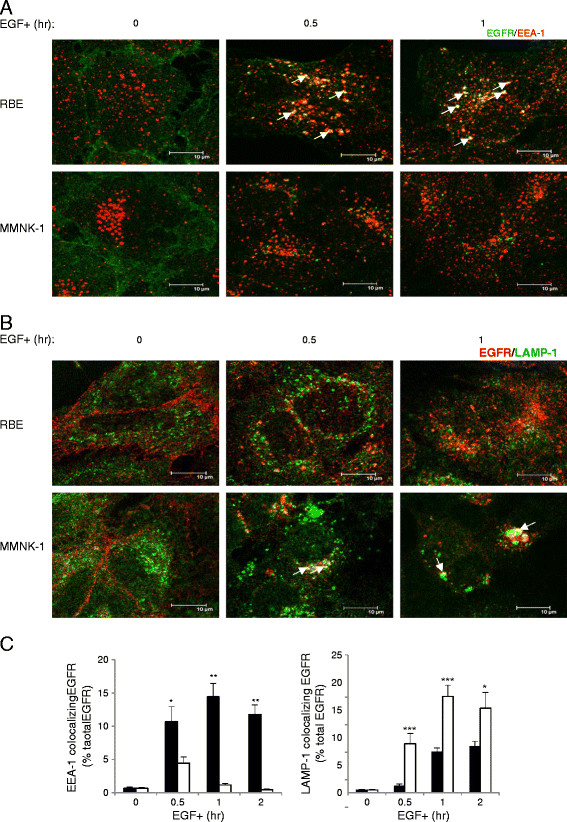
**Post-endocytic trafficking of EGFR in RBE and MMNK-1 cells.** (**A**) Distribution of EGFR (Alexa Fluor 488) and Early endosome antigen 1 (EEA-1) (Alexa Fluor 555) in RBE and MMNK-1 cells before and after 0.5 and 1 hr of EGF stimulation as obtained by immunofluorescence. White arrows show points of colocalization. Scale bars: 10 μm. (**B**) Distribution of EGFR (Alexa Fluor 555) and Lysosomal-associated membrane protein-1 (LAMP-1) (Alexa Fluor 488) in RBE and MMNK-1 cells before and after 0.5 and 1 hr of EGF stimulation as obtained by immunofluorescence. White arrows show points of colocalization. Scale bars: 10 μm. (**C**) Left: colocalization rate between EGFR and EEA-1 in RBE cells (black columns) and MMNK-1 cells (white columns) before and after 0.5, 1, and 2 hr of EGF stimulation. Right: colocalization rate between EGFR and LAMP-1 in RBE cells (black columns) and MMNK-1 cells (white columns) before and after 0.5, 1, and 2 hr of EGF stimulation. Values are the percentage of the integrated density of EEA-1 or LAMP-1-colocalizing EGFR compared to that of total EGFR. *p < 0.05; **p < 0.01; ***p < 0.001. n = 10 fields. Three independent experiments were performed.

We next investigated the route of endocytosed EGFR for trafficking to lysosomes and degradation by immunostaining for EGFR and Early Endosome Antigen 1 (EEA-1), a marker of early/sorting endosomes (Figure 3A), or for EGFR and Lysosomal-Associated Membrane Protein 1 (LAMP-1), a lysosome marker (Figure 3B). The colocalization rate was calculated as the percentage of the integrated density of endosome/lysosome marker-colocalizing EGFR compared with that of total EGFR (% total EGFR) (Figure 3C). Double staining of EGFR and EEA-1 showed that EGFR remained colocalized with EEA-1 in RBE cells, but not in MMNK-1 cells, after 30 min of EGF stimulation (Figure 3A). Colocalization rate calculations confirmed that EEA-1-colocalizing EGFR was greater in RBE cells than in MMNK-1 cells after both 30 min (10.7 ± 2.2% vs. 4.4 ± 0.9% total EGFR) (p < 0.05, n = 10, Figure 3C, left) and 1 hr (14.4 ± 2.0% vs. 1.2 ± 0.2% total EGFR) (p < 0.01, n = 10, Figure 3C, left) of EGF stimulation. Double staining of EGFR and LAMP-1 showed that EGFR did not colocalize with LAMP-1 in RBE cells, but rather aggregated near the nucleus and colocalized with LAMP-1 in MMNK-1 cells after 30 min of EGF stimulation (Figure 3B). Colocalization rate calculations verified that LAMP-1-colocalizing EGFR was markedly less in RBE cells than in MMNK-1 cells after 30 min (1.3 ± 0.3% vs. 8.9 ± 1.9% total EGFR) (p < 0.001, n = 10, Figure 3C, right) and 1 hr (7.5 ± 0.7% vs. 17.5 ± 2.0% total EGFR) (p < 0.001, n = 10, Figure 3C, right) of EGF stimulation. These results demonstrated that whereas EGFR was retained in early endosomes upon EGF stimulation in RBE cells, it was sorted to lysosomes quickly in MMNK-1 cells.

### Recycling of EGFR was enhanced in RBE cells

**Figure 4:**
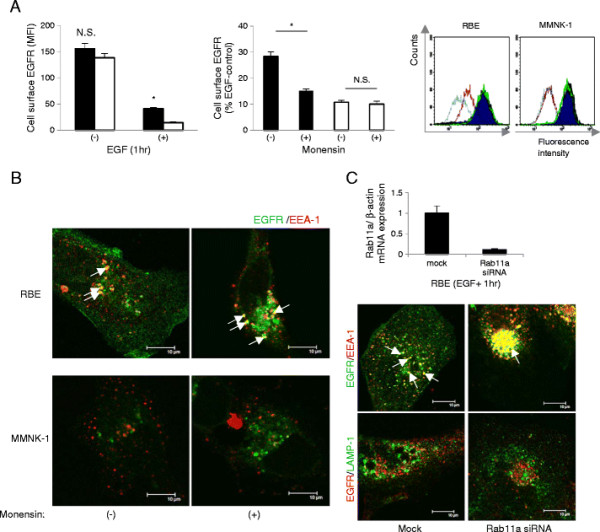
**Effect of recycling inhibition on cell surface EGFR upon EGF stimulation in RBE and MMNK-1 cells.** (**A**) Left: cell surface EGFR before and after 1 hr of EGF stimulation without monensin treatment. Black columns: RBE; white columns: MMNK-1. Values are presented as the Mean Fluorescence Intensity (MFI) of cell surface EGFR.*p < 0.05. Middle: cell surface EGFR after 1 hr of EGF stimulation without or with monensin in RBE cells (black columns) and MMNK-1 cells (white columns). Values are presented as the percentage of the MFI of cell surface EGFR compared to that of RBE or MMNK-1 cells before EGF stimulation. *p < 0.05. Right: representative FACS data. Dark blue: cell surface EGFR before EGF stimulation without monensin; purple: cell surface EGFR after 1 hr of EGF stimulation without monensin; green: cell surface EGFR before EGF stimulation with monensin; light blue: cell surface EGFR after 1 hr of EGF stimulation with monensin. (**B**) EGFR (Alexa Fluor 488) and EEA-1 (Alexa Fluor 555) distribution after 1 hr of EGF stimulation without or with monensin in RBE and MMNK-1 cells as obtained by immunofluorescence. White arrows show points of colocalization. Scale bars: 10 μm. (**C**) Upper: suppression of Rab11a expression by siRNA. Lower: EGFR (Alexa Fluor 488) and EEA-1 (Alexa Fluor 555) distribution or EGFR (Alexa Fluor 555) and LAMP-1 (Alexa Fluor 488) distribution after 1 hr of EGF stimulation in mock- or Rab11a siRNA-transfected RBE cells as obtained by immunofluorescence. White arrows show points of colocalization. Scale bars: 10 μm. All results are representative of three or more independent experiments.

As endocytosed EGFR were not sorted to lysosomes following sequestration in early endosomes in RBE cells, we hypothesized that non-degraded EGFR might undergo recycling back to the cell membrane after this trafficking block. We first quantified cell surface EGFR before and after EGF stimulation. Before EGF stimulation, the amount of cell surface EGFR was comparable between RBE and MMNK-1 cells. After 1 hr of EGF stimulation, the amount of EGFR on the surface of RBE cells (41.1 ± 2.2MFI) was approximately three times that on MMNK-1 cells (14.6 ± 1.2MFI) (p < 0.05, n = 4, Figure 4A, left). We then investigated if the excessive EGFR on RBE cell surfaces was caused either by slow endocytosis or enhanced recycling by employing the recycling inhibitor monensin. The percentage values of cell surface EGFR after EGF stimulation compared to those beforehand (% control) were compared between groups with or without monensin treatment for both cell lines. In RBE cells, monensin treatment significantly reduced cell surface EGFR expression (15.1 ± 0.8% vs. 28.3 ± 1.8% control) after 1 hr of EGF stimulation (p < 0.05, n = 4, Figure 4A, middle). However, no difference was observed between groups with or without monensin treatment in MMNK-1 cells (Figure 4A, right). These results indicated that approximately half of the excess EGFR expression on RBE cell surfaces was due to abnormal recycling. Immunofluorescent staining of EGFR and EEA-1 showed that monensin treatment also retained more EGFR in early endosomes in RBE cells (Figure 4B). Immunofluorescent staining of EGFR and EEA-1 or LAMP-1 demonstrated that Rab11a depletion could suppress cell surface EGFR expression and maintain more EGFR in the early endosome of RBE cells (Figure 4C, lower panel, upper right). Rab11a depletion could not promote EGFR sorting into late endosome/lysosome in RBE cells (Figure 4C, lower panel, lower right), hence EGFR degradation was not enhanced by this treatment (data not shown).

### EGFR was hypo-ubiquitinated, EGFR-c-Cbl association was impaired, and Tyr1045 was hypo-phosphorylated upon EGF stimulation in RBE cells

**Figure 5:**
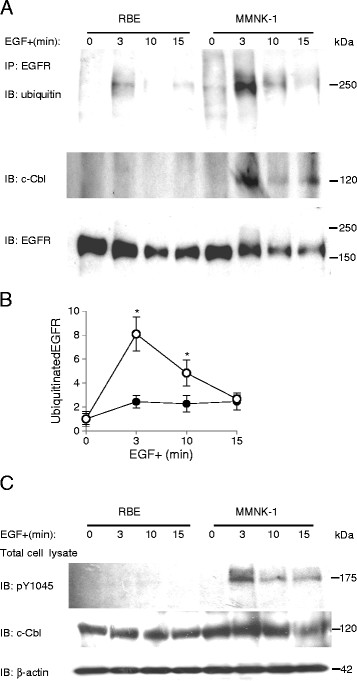
**EGFR ubiquitination, c-Cbl/EGFR association, and pY1045 expression upon EGF stimulation in RBE and MMNK-1 cells.** (**A**) EGFR ubiquitination and EGFR-associated c-Cbl before and after 3, 10, and 15 min of EGF stimulation. (**B**) Quantification of EGFR ubiquitination from Western blotting in RBE cells (closed circles) and MMNK-1 cells (open circles). Values are standardized to the optical intensity of EGFR and presented as values relative to that of ubiquitinated EGFR/total EGFR in RBE or MMNK-1 cells before EGF stimulation. *p < 0.05. (**C**) Expression of pY1045 and c-Cbl in total cell lysates before and after 3, 10, and 15 min of EGF stimulation. All results are representative of three or more independent experiments.

To explore the mechanism of impaired EGFR sorting for degradation in lysosomes in RBE cells, we compared levels of EGFR ubiquitination following 3, 10, and 15 min of EGF stimulation between RBE and MMNK-1 cells. Ubiquitination of EGFR in RBE cells remained low during the 15-min time course, whereas in MMNK-1 cells, ubiquitinated EGFR peaked at 3 min after EGF stimulation, and then gradually decreased (Figure 5A, B). The difference of EGFR ubiquitination was most remarkable after 3 min of EGF stimulation between the cell lines (2.4 ± 0.5 vs. 8.1 ± 1.4) (p < 0.05, n = 4, Figure 5B). Also, we studied the association between EGFR and c-Cbl before and after EGF stimulation. C-Cbl associated with EGFR after 3, 10 and 15 min of EGF stimulation in MMNK-1 cells although this phenomenon was not observed in RBE cells (Figure 5A). When we studied the expression of phosphorylated Tyr1045 (pY1045) and total c-Cbl after 3, 10 and 15 min of EGF stimulation in both cell lines, we uncovered that phosphorylation of Tyr1045 was impaired in RBE cells but intact in MMNK-1 cells (Figure 5C).

## Discussion

The present study indicated that gene and protein expression levels of EGFR before EGF stimulation did not differ significantly between RBE and MMNK-1 cell lines. However, whereas EGFR protein expression was markedly down-regulated under sustained EGF stimulation in MMNK-1 cells, a considerable amount of endocytosed EGFR was retained in early endosomes in RBE cells. EGFR over-expression on the plasma membrane was observed in this cell line as well.

Clathrin-coated vesicles containing EGF-EGFR complexes release their coat and fuse with early endosomes quickly following endocytosis. Since the EGF-EGFR complexes do not dissociate in the early endosome, EGFR remains phosphorylated and associated with Cbl [29]. Cbl mediates the interaction between EGFR and endosomal sorting complex required for transport (ESCRT) machinery and promotes EGFR sorting in multi-vesicular bodies (MVB) [30]. An early dissociation of the EGF-EGFR complex because of abnormally low pH in endosomes or an unstable association between EGF and EGFR-HER2 heterodimers enhances the recycling of unoccupied EGFR [10]. In our study, EGFR was retained in early endosomes and kept phosphorylated at Tyr1068 in RBE cells. Thus, early dissociation of the EGF-EGFR complex was not considered to be the mechanism of impaired degradation and enhanced recycling of EGFR in RBE cells.

Cbl-mediated ubiquitination is critical for EGFR early endosome exit, lysosome sorting, and degradation [18,19]. Since low levels of EGFR ubiquitination and poor Cbl association were observed in RBE cells, we considered these factors to be attributed to the dramatically diminished EGFR degradation in RBE cells. Reduced Cbl association and/or impaired EGFR ubiquitination could be linked with a Cbl mutation in the RING Finger domain, especially cysteine 381 (C381), which is the first cysteine of the C_3_HC_4_ zinc finger motif [31], or at the RING finger C-terminal flank, especially valine 431 (V431) and phenylalanine 434 (F434) [20]. Apart from mutations in c-Cbl, the loss of the Cbl docking site on EGFR (pY1068 and pY1045) for numerous reasons, such as EGFR mutations in the tyrosine kinase domain [32,33] or mutations at ubiquitination sites of EGFR [34], could also lead to reduced Cbl association and/or impaired EGFR ubiquitination. In RBE cells, no mutations at C381, V431 or F434 of Cbl, the tyrosine kinase domain or Tyr1068 or 1045 residues of EGFR, or of the extracellular domain that binds to ligands (exon 2 ~ 16) [35] were identified. Meanwhile, we observed that transfected wtEGFR behaved similarly to endogenous EGFR; Myc-tagged wtEGFR in pcDNA3.1, a kind gift from Dr. Tokuzou Arao [36], was transfected into RBE cells and revealed that Myc-tagged wtEGFR was retained in early endosomes and was not sorted into late endosomes/lysosomes (data not shown). This verified that endogenous EGFR was not the reason for impaired EGFR degradation in RBE cells. However, unlike Tyr1068 that could be phosphorylated normally, Tyr1045 could not be phosphorylated following EGF stimulation. Combining this with the data from Grøvdal et al. [37], who described that a direct association of c-Cbl with EGFR pY1045 was important for MVB sorting of EGFR, we surmised that aberrant EGFR sorting into lysosomes in RBE cells was caused by an impaired association between c-Cbl and EGFR through pY1045.

Hypophosphorylation of Tyr1045 has been reported in non-small cell lung cancers (NSCLCs) bearing EGFR mutations in the tyrosine kinase domain [33] and in an EGFRvIII variant bearing an internal in-frame deletion in the extracellular domain [38]. However, no EGFR mutation was identified in our RBE cells, nor was Tyr1045 of the transfected wtEGFR seen to be phosphorylated (data not shown). Willmarth et al. [39] reported similar findings caused by stimulation with one member of the EGF family, Amphiregulin (AR), in SUM149 human breast cancer cells. In their study, AR activation of EGFR resulted in increased steady-state levels of the receptor that accumulated at the cell surface as a result of decreased phosphorylation of Tyr1045 on EGFR (wild type) and a resultant failure to ubiquitinate [39]. However, the mechanism of Tyr1045 hypophosporylation without mutation as in RBE cells remains unclear. Apart from an abnormality of EGFR or its ligand, a diminished EGF-EGFR interaction affinity and reduced receptor-associated tyrosine kinase activity caused by phosphorylation of EGFR threonine residues through protein kinase C (PKC)-dependent [40] or independent pathways [41] may be speculated as the mechanism of Tyr1045 hypophosphorylation in RBE cells. Uncovering the mechanism of Tyr1045 hypophosphorylation is of great importance in restoring EGFR degradation and negatively controlling EGFR over-activation in RBE cells.

Lastly, we employed two methods to verify the role of EGFR recycling in cell membrane EGFR over-expression: monensin treatment [24,42] and Rab11a protein depletion [43]. Rab proteins regulate various steps in recycling: Rab4 regulates fast/direct recycling from the early endosome to the cell membrane [44], and Rab11a regulates recycling from the deeper perinuclear recycling compartment [43]. Monensin treatment blocks recycling from both the early endosome and perinuclear recycling compartment [24]. Suppressed cell surface EGFR expression by monensin treatment or Rab11a depletion indicated that enhanced recycling occurred at least in the perinuclear recycling compartment in RBE cells with EGF stimulation. Furthermore, early endosome retainment of EGFR without recycling inhibition showed that recycling in RBE cells did not significantly take place through fast/direct recycling from early endosomes.

## Conclusions

Recent strategies examining EGFR-targeted therapy of cholangiocarcinoma have focused on EGFR tyrosine kinases related to the signaling of mutated and over-expressed EGFR. Our results indicated significantly impaired EGFR degradation associated with hypo-phosphorylation of Tyr1045 and enhanced recycling of EGFR to the cell membrane in RBE cells. In cholangiocarcinoma cell types resembling RBE, up-regulation of EGFR Tyr1045 phosphorylation may be a potentially useful molecular alteration in EGFR-targeted therapy.

## Competing interests

The authors declare that they have no competing interests.

## Authors’ contributions

AG and AK designed the study and drafted the manuscript. AG carried out the studies under the instructions for HM in direct cDNA sequence analysis, MK for flow cytometry study, and MT for Western blot analysis and RNA interference study. MT and SM critically read and revised the manuscript. All authors read and approved the final manuscript.

## Pre-publication history

The pre-publication history for this paper can be accessed here:

http://www.biomedcentral.com/1471-2407/12/179/prepub
